# Domestic pet dogs (*Canis lupus familiaris*) do not show a preference to contrafreeload, but are willing

**DOI:** 10.1038/s41598-024-51663-x

**Published:** 2024-01-15

**Authors:** Liza Rothkoff, Lynna Feng, Sarah-Elizabeth Byosiere

**Affiliations:** 1 Thinking Dog Center, Department of Psychology, Hunter College, City University of New York, New York, NY 10065 USA; 2 Guide Dogs for the Blind, San Rafael, CA 94903 USA

**Keywords:** Animal behaviour, Psychology

## Abstract

Contrafreeloading is the behavior of working for food when also provided with identical food that does not require effort to obtain. This behavior has been observed in humans and non-human animals, including domesticated species. In the current investigation, we assessed whether companion animals, specifically domestic dogs, contrafreeload when presented with two feeders simultaneously, a snuffle mat (a work for food device) and a tray (a freely available food device). Thirty-eight pet dogs participated in the study in which ten feeding trials were presented where food was distributed equally in both feeders. Three overarching research questions were considered: (1) Do dogs prefer to contrafreeload and/or are they willing to contrafreeload, (2) is activity (step count) or body condition score (BCS) related to contrafreeloading behavior and, (3) does previous experience with puzzle feeders impact contrafreeloading behavior? Two general linear models were conducted assessing the effects of sex, experience, age, activity and BCS on the proportion of first choices to the snuffle mat (*IC*_*s*_) and the number of interactions with the snuffle mat while food was still available in the tray (*IN*_*s*_)_*.*_ Overall, when assessing the proportion of first choices to each feeder, dogs demonstrated a *willingness* to contrafreeload but not a *preference* to contrafreeload. In a reduced model, only one term, owner-reported body condition score, had a significant effect, suggesting that dogs with a higher BCS demonstrated a greater proportion of first choices for the snuffle mat (*F* (1,36) = 7.72, *p* = 0.009, η^2^p = 0.177). In evaluating the number of interactions with the snuffle mat while food was still available in the tray, the model was not significant (*F* (5,29) = 1.231, *p* = 0.320, η^2^p = 0.175). This study represents the first investigation of contrafreeloading in domestic pet dogs and informs recommendations for canine enrichment.

## Introduction

Contrafreeloading is the behavior of working for a resource that requires effort to obtain when also provided with a similar resource that is freely available^1–3^. Historically, this resource has been food, although contrafreeloading for water and other resources has also been studied [e.g.,^4^]. The presence of contrafreeloading behavior is particularly interesting given that it seems to oppose a basic tenet of animal learning and evolutionary theory, as well as optimal foraging theory^1^—that animals should maximize rewards and minimize costs^5,6^. Inglis et al.^1^ suggests a functional explanation as to why this stimulus may be reinforcing: there is an advantage to animals to gather information in changing environments through the information primacy model.

The information primacy model suggests that information gathering is of utmost importance to animals living in uncertain environments, as it is essential for animals to reduce environmental uncertainties^7^. Additionally, animals might not just be working for food, but could also be looking for information regarding future food sources, known as the “information hypothesis”^8^. Therefore, contrafreeloading is less likely to occur in animals whose access to food is not dependent on searching for unpredictable food sources^1^. Consistent with this hypothesis, research has shown contrafreeloading behavior may represent an adaptive form of exploration^9^ by allowing an individual to gain information about their environment^1^. Thus, it is of particular importance to evaluate overall activity when studying contrafreeloading^10^, as animals could be active when exploring their environments.

Various factors have been found to affect the presence and strength of contrafreeloading behavior such as training history (animals with more experience on a work for food task show more contrafreeloading), effort needed to obtain food (work that requires more effort results in less contrafreeloading), rearing conditions (sensory deprivation increases contrafreeloading), and hunger (food deprivation reduces contrafreeloading)^1^. Manipulation of the environment may also drive contrafreeloading behavior^1^, as being able to control one’s environment is a critical trait to the survival of an individual animal^11^. This theory suggests that working for a food source involves more environmental manipulation than eating freely available food^1^. For example, rats have been found to prefer pressing a lever (environmental manipulation) for food over response-independent food delivered at the same rate^12,13^. Additionally, domestication is also thought to influence an animal’s level of contrafreeloading behavior. For example, domestic White Leghorn chickens (bred for egg-laying) show less contrafreeloading and less exploration than their ancestor, the red jungle fowl^14,15^, possibly because in natural conditions, wild animals often live in uncertain environments, therefore increasing the need to explore the environment. The ability to provide an animal with the opportunity to display its natural abilities and tendencies can be a challenge in captive animal welfare^16^. Some of these needs, such as appetitive feeding behaviors, could be fulfilled by providing feeding enrichment activities to animals^17^. Many studies on contrafreeloading have been assessed through foraging behavior [e.g.,^9,18^], as many of the species tested are natural foragers^1^.

To date, contrafreeloading has been functionally defined in multiple ways. Some studies have evaluated contrafreeloading as a traditional two-choice preference to approach a feeder that requires effort and one that does not [e.g.,^19^]. Others have measured contrafreeloading as consuming more food from a feeder that requires effort than the one that does not (e.g.,^10,20^], or by spending more time in proximity to the effortful feeder than the free feeder [e.g.,^9^]. Still some argue that contrafreeloading should be measured as a *willingness* to work for any food while free food is available^9^, since optimal foraging theory would suggest optimal effort would be to consume all freely available food before working for the same resource. While not all studies show animals demonstrating a clear *preference* to contrafreeload [e.g.,^2,21^; but not^9,22^], willingness has been observed in nearly all species studied [e.g.,^23,24^]. Thus, it is important to consider assessing both preference and willingness when investigating contrafreeloading behavior, as well as the need to evaluate individual subject differences and their environment. For an overview of the current peer-reviewed literature in which contrafreeloading is assessed in animals, emphasizing which species show a *preference* and/or a *willingness* to contrafreeload, see Table 1.

**Table 1:** Overview of food contrafreeloading studies in which species, domestication status, preference and willingness to contrafreeload are identified.

Species	Domesticated?	Preference for CFL?	Willingness to CFL?
Starlings (*Sturnus vulgaris*)^8,20^	No	Yes^8,20^	Yes^8,20^
Red jungle fowl (*Gallus gallus*)^14,15^	No	Yes^14,15^	Yes^14,15^
Grizzly bears (*Ursus arctos horribilis*)^9^	No	Not assessed	Yes
Maned wolves (*Chrysocyon brachyurus*)^22^	No	Not assessed	Yes
Rhesus macaques (*Macaca mulatta*)^23^	No	Not assessed	Yes
Japanese macaques (*Macaca fuscata*)^25^	No	Not assessed	Yes
Stump-tailed macaque (*Macaca arctoides*)^26^	No	Not assessed	Yes
Chimpanzees (*Pan troglodytes*)^27^	No	Not assessed	Yes
Kea parrots (*Nestor notabilis*)^28^	No	Yes	Yes
Grey parrots (*Psittacus erithacus*)^29^	No	Yes	Yes
Crows (*Corvus brachyrhynchos*)^30^	No	Yes	Yes
Giraffes (*Giraffa cameloparadalis*)^31^	No	Yes (with individual variation)	Yes
Common rats (*Rattus norvegicus*)^2,12,13,32–36^	Yes	Yes^2,12,32,33,35^ No^13,34^ Not assessed^36^	Yes^2,12,13,32–36^ Not assessed^34^
Pigs (*Sus scrofa*)^18,37^	Yes	Yes^18^ No^37^	Yes^18,37^
Goats (*Capra hircus*)^19^	Yes	Not assessed	Yes
Domestic cattle (*Bos taurus*)^24^	Yes	Not assessed	Yes
Domestic fowl (*Gallus gallus domesticus*)^14,15,38–42^	Yes	Yes^38,39^ No^14,15,41^ Not assessed^40^	Yes^14,15,38–42^
Pigeons (*Columbia livia domestica*)^36,43,44^	Yes	Not assessed^36,43,44^	Yes^36,43,44^
Siamese fighting fish (*Betta splendens*)^45^	Yes	Yes	Yes
Domestic cats (*Felis catus*)^10,46^	Yes	No^10,46^	Yes^10^ No^46^
Humans (*Homo sapiens*)^12,21,47^	N/A	Yes^12,21,47^	Yes^12,21,47^

Animals who show a preference to contrafreeload are also presumed to have a willingness to contrafreeload.

From the available literature, domestic cats’ contrafreeloading behavior stands out compared to other species tested. Even though most animals have demonstrated a preference to contrafreeload, recently one companion animal, the domestic cat, does not^10,46^. Koffer and Coulson^46^ reported that none of the six laboratory-housed cats in their study demonstrated any *willingness* to contrafreeload; all six cats tested ate all freely available food before working for food. In a more recent investigation of cat contrafreeloading behavior, Delgado et al.^10^ observed that five of the 17 pet cats chose to work for food before eating freely available food at least once, however, none met criteria to be considered as having a *preference* for contrafreeloading. Since searching for unpredictable food sources is typically not a part of the daily life of a domestic cat, it may be that they are unlikely to contrafreeload due to their domestication history.

But, the question remains, why might cats show weaker contrafreeloading behavior^10^ than other species? Could it be due to their socio-ecological environment? The cat population studied by Delgado et al.^10^ consisted of indoor cats living a companion animal lifestyle, which may have a closer bond to humans and less environmental uncertainty than other animals studied. To evaluate if companion animal lifestyle results in weaker contrafreeloading behavior, the next logical candidate in which to explore the behavior of contrafreeloading is the domestic dog (*Canis lupus familiaris*) (hereafter referred to as dog). To our knowledge no prior peer-reviewed study has evaluated contrafreeloading behavior in dogs. In terms of their eating and feeding behavior, pet dog hunting behavior has been substantially genetically modified throughout the domestication process^48^. Feral dogs demonstrate scavenging behaviors^49^; for free-ranging dogs, sampling of food plays an important role in foraging behaviors and the selection of food^50^. This behavior is markedly different in pet dogs, where the selection of food is based on odor, flavor, texture, and appearance^51^. Many owners of pet dogs give their dogs feeding enrichment such as food enrichment toys that mimic natural foraging opportunities and have been suggested to improve pet dog welfare^52^.

Many pet dog owners offer their dogs a foraging enrichment device called a “snuffle mat,” wherein food is hidden within fabric, encouraging dogs to use their noses to search for food. Providing dogs with opportunities to channel their energies into using their noses in scent activities, such as by providing a snuffle mat, is posited to help anxious dogs build confidence, or help excited dogs calm down with a mentally tiring activity^53^. Prior research reported that the welfare of a dog is improved when given more opportunities and time to forage^52^. The impact of feeding enrichment has been studied in kenneled dogs [e.g.,^17^], and the benefits of enrichment to dogs in the home setting have been documented^54^. Providing food enrichment to dogs can result in an increase of time spent on appetitive feeding behaviors, allowing dogs to satisfy their natural tendencies, which is inherently rewarding^17^. Feeding enrichment has also been shown to result in a higher activity level^17^, which can have beneficial effects such as preventing obesity^55,56^. However, there remains a gap in the literature of whether dogs contrafreeload. In other words, would dogs choose to engage in this foraging behavior when the food is also freely available? Given previous literature, we aimed to evaluate if dogs contrafreeload. Dogs were presented with two feeding devices, a tray (freely available food) and a snuffle mat (work for food device), to investigate the following research questions:

1. Do dogs contrafreeload?
  - Do dogs *prefer* to contrafreeload, as measured by a first-choice preference test between the tray and snuffle mat?
  - Are dogs *willing* to contrafreeload, as measured by the presence of approaching and interaction with the snuffle mat before finishing the food in the tray?
2. Is physical fitness related to contrafreeloading behavior in dogs?
  - Is contrafreeloading behavior affected by activity level as measured by average daily step count?
  - Is contrafreeloading behavior impacted by owner-reported body condition score (BCS)?
3. Is contrafreeloading behavior related to previous experience(s) with puzzle feeders?

## Methods

The present study sought to adapt the methods of Delgado et al.^10^ as a community science project to assess a dog’s *preference* and *willingness* to contrafreeload. This study was preregistered using AsPredicted (#102462) and received ethics approval from the CUNY Hunter College IACUC on March 9, 2022 (SEB-FreeloadingDogs 2/25). The study followed ARRIVE guidelines and was carried out in accordance with the relevant guidelines and regulations. Data collection occurred from June 2022 to November 2022.

### Participants

Dogs and their owners were recruited through social media (e.g., Instagram and Facebook) from posts encouraging participation in the study, as well as through the Thinking Dog Center’s (TDC), a dog cognition and behavior research group, database of dogs. Dog owners volunteered their dogs’ participation in the study and filled out an initial Qualtrics survey to determine eligibility. Prior to participating in the study, dog owners signed a consent form, in which the owners agreed to the data they submitted to the researchers being used in a publication, thesis, or presented at conferences. Owners were also able to withdraw their dogs’ data from the study at any time, up to 4 weeks following the completion of their dogs’ participation in the project. No owner behavior was behaviorally coded and no identifying information for the owners was used for research purposes.

In addition to basic demographic information, owners were asked how often the dog is fed, what type of food the dog eats (wet/dry/both), and the amount of food that the dog eats in cups. To be eligible for the study, dogs had to be fed dry food, had to be at least one year old, and had to not have any known visual impairments. Research has demonstrated that prior experience with an earned reinforcer can increase contrafreeloading behavior [e.g.^43^] and increased hunger reduces contrafreeloading behaviors^8,13^. As such, additional questions regarding food motivation, experience with food enrichment toys, and the dog’s weight and body condition score (BCS) were asked via the survey (the survey, in its entirety, can be found in Supplementary Material S1).

A total of 40 dogs were recruited to participate in the study, however, the final sample of dogs consisted of 38 domestic pet dogs (22 female, 16 male) between 1 and 11 years old (M = 4.21, SD = 2.89) with 17 purebred dogs and 21 mixed breed dogs. Two dogs did not meet study inclusion requirements in the acclimation sessions (dogs were required to eat from both feeders in the acclimation trials) and were excluded from further participation. One dog was excluded as they did not eat at all during the acclimation trials, and the other dog excluded only ate from the tray. Dogs who successfully ate from both the tray and the snuffle mat were exposed to the testing protocol (*n* = 38).

### Study materials

The researchers provided the owners with all materials needed to conduct the study in their homes. Dog owners were based in the New York City and Tri-State area as these materials were picked up from the TDC. One dog (Barley) was in California; therefore, his supplies were mailed to the owner. Owners were asked to come to the TDC once (142 West 36th Street New York, New York 10018) during a predetermined time frame. This in-person meeting allowed the researchers to explain the study to the owners step-by-step, and answer any questions they may have had. The study materials provided consisted of a tray (freely available feeding device), a snuffle mat (work for food feeder), a Fi Series 2 Collar (https://tryfi.com/), a scale, floor stickers, instructions (Supplementary Material S2), and a data sheet (Supplementary Material S3). The tray was a 12 in diameter black plastic plate. The snuffle mat was created by the research team using cut up pieces of fleece woven through holes of anti-fatigue mats. The mats were cut into a circular shape with a 12 in diameter to match the size of the tray. Researchers also adhered a Dycem non-slip material to the bottom of both feeders to minimize any sliding movement of the feeders during feeding trials. Given that previous research suggests there may be a relationship between exploration and contrafreeloading^20^, we asked owners to place a smart collar (Fi Collar) on their dog for the duration of the study period. This allowed the researchers to measure the number of steps the dog took each day throughout the study. In addition to the materials provided, owners also needed to provide the dog with enough dry food (e.g., kibble) for one full meal during each feeding session and use their own camera-enabled device to record the sessions.

### Acclimation trials

Dogs were first acclimated to the snuffle mat, the tray, and the activity tracker over a span of four acclimation feedings. The activity tracker was placed on the dog prior to the first acclimation feeding. However, one dog (Pogacs) had six acclimation feedings due to food measurement errors during the first two acclimation trials. All acclimation sessions were filmed and sent to the research team.

For the first acclimation feeding, all dogs were provided with 25% of their regular meal intake in the snuffle mat and 75% of their mealtime rations on the tray. For the second and third acclimation feedings, all the dog’s daily food intake was split evenly between the tray and the snuffle mat. For the fourth and final acclimation feeding, all dogs were provided with 75% of their daily food intake in the snuffle mat and 25% of their daily intake on the tray. The gradual proportional change of food in each feeder presented during the acclimation sessions ensured that dogs included in the study ate from both feeders and is in line with the methods presented in Delgado et al.^10^.

### Test trials

Once a dog successfully completed all acclimation sessions, the test sessions began. In line with Delgado et al.^10^, owners were instructed to place food (50% of their dog’s meal intake) in the snuffle mat and the same amount of food in the similarly sized tray (just as was done in Acclimation Sessions 2 and 3). The researchers provided the owners with the exact measurements of food the dog should receive for each trial on the data sheet (presented in grams) based on the dog’s mealtime ration. We requested owners present the feedings 1–2 times a day over 5–10 days depending on the dog’s usual mealtime feeding schedule. This allowed for 10 trials, or feedings, per dog, which paralleled the 10 trials observed in Delgado et al.^10^. We did not specify the time of the feedings, or the time in between feedings to minimize any disruption to the dog’s daily routine. Owners were asked to weigh the food in grams on a provided scale before giving it to their dogs and record this value on the data sheet. One dog (Nessie) completed 11 trials due to food measurement errors.

**Figure 1:**
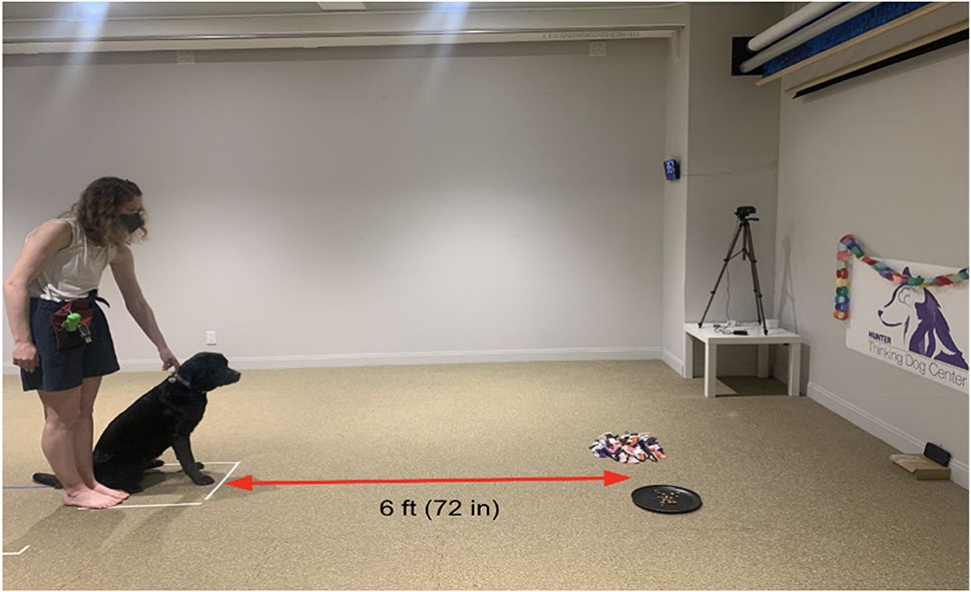
Depiction of room setup when the dog is ready to be released to eat, showing the dog’s distance from the feeders.

Owners were asked to place the tray and snuffle mat two feet (24 inches) apart from each other on the floor, and equidistant from the dog. Owners then placed their recording device four feet away from the dog and began video recording. The owner would then bring the dog to the starting position (six feet away from the feeders) and release the dog (see Fig. 1 for a depiction of the typical study design setup). Owners were asked to move away from the feeders once placed on the ground and ignore the dog while they ate so as to avoid unintentionally cuing them. The owner stopped the recording when the dog walked away from and stopped interacting with both feeders for a minimum of 10 s. Dogs were given a maximum of 15 min to eat from the snuffle mat and/or tray for each trial. Just as in the acclimation sessions, all test sessions were filmed and sent to the researcher team.

The owners were also asked to record the date and time each trial started. Following each trial, the owner then was asked to pick up the feeders and separately use the provided scale and weigh the food (in grams) left in each feeder if there was any food leftover and record this value on the respective test’s entry on the data sheet. Both feeders were removed from the floor when the trial session ended. Any remaining food was weighed, recorded, and then offered to the dog in their regular feeding bowl.

The left/right placement for the freely available food tray and the puzzle feeder were randomized and counterbalanced through a predetermined pseudorandomized sequence (random.org) for both the acclimation and test trials. The researchers created two versions of the data sheet, each with a different left/right sequence. Nineteen of the dogs received the first version of the data sheet, and the other 19 dogs received the second version of the data sheet. Most dogs were shown the left and right placements for the feeders an equal number of times, with no more than three of the same placements in a row. However, four dogs did not have the feeder placement counterbalanced during the test sessions due to owner error. In these cases, a dog may have had four placements in a row. We determined that one dog had a side bias for the right (Toby), determined by choosing the right side 100% of the time across the ten test trials.

### Video submission and coding

Video footage from the feedings was sent to the research team along with a copy of the completed data sheet upon completion of the study. Post-submission, all videos were coded through BORIS (Behavioral Observation Research Interactive Software)^57^ to evaluate the duration of time each dog interacted with either the tray or the snuffle mat, as well as to record which feeder the dog approached first. First approach was coded when the dog began sniffing, eating, or touching the feeder. Consistent with Delgado et al.^10^, sniffing, eating, and touching the feeder were consolidated into one measure of interacting with either the tray or snuffle mat since it was not always clear how the dog was interacting with the feeder.

### Data analysis

All data analyses were conducted in Jamovi. Three coders were trained on a subset of trial videos for eight dogs (80 videos total), ~ 21% of the sample. Inter-rater reliability, or agreement between the coders, on identifying the binary first-choice location (i.e., the snuffle mat or the tray) was 100%. Inter-rater reliability for interaction with the snuffle mat while food was left in the tray was calculated in AgreeStat^®^ 360 using a Gwet’s AC1, with κ = 0.962 (95% CI, 0.94 to 0.98), *p* < 0.001.

Following Delgado et al.^10^, we scored dogs’ initial choice (proportion of trials choosing the snuffle over the tray: *IC*_*s*_) prior to any food being consumed. To evaluate our first research questions, do dogs prefer to contrafreeload and are dogs willing to contrafreeload, a two-tailed binomial test was conducted. The proportion of first choices to the snuffle mat (*IC*_*s*_) was used as a measure of *preference* for contrafreeloading. Based on a two-tailed binomial test (*p* = 0.025), we defined dogs with an *IC*_*s*_ score equivalent or higher than 0.8 as showing a significant preference to contrafreeload. Conversely, we defined dogs with *IC*_*s*_ scores less than or equal to 0.2 as demonstrating a significant preference to freeload (i.e., preference to initially choose the tray). Dogs were defined as *willing* to contrafreeload if their *IC*_*s*_ score was greater than 0. We chose these definitions in alignment with the suggestion from McGowan et al.^9^ to acknowledge any level of effort displayed to acquire earned resources in the presence of free resources.

During video analysis, we identified that dogs demonstrated notable switching behavior between the two feeders within a trial. Following the definition of contrafreeloading as any demonstration of working for food while free food remains available, we added an additional measure, *IN*_*s*_, defined as the number of interactions with the snuffle mat (minimum of 2 s) while food was still in the tray. To avoid including interactions with the snuffle mat when no food was left in the tray, interactions with the snuffle mat were only included if they were followed by a subsequent interaction with the tray (minimum of 2 s).

To investigate research questions two and three, whether physical fitness and previous experience with enrichment devices impacted contrafreeloading behavior, two general linear models (GLM) were conducted utilizing the GAMLj package in Jamovi. The first model assessed the effects of sex, age, activity level, BCS and prior experience with enrichment devices on *IC*_*s*_:

$${IC }_{s}\sim 1+Sex+Experience+Age+Activity+BCS$$

The second model assessed the effects of sex, age, activity level, BCS and prior experience with enrichment devices on *IN*_*s*_:

$${IN }_{s} \sim 1+Sex+Experience+Age+Activity+BCS$$

## Results

Fourteen of the 38 dogs consumed all the food presented in both feeders during every trial. For a detailed overview of each dog’s data, see Table 2.

**Table 2:** Descriptive overview of the individual results. The table shows all data for every participant. *E*_*s*_ and *E*_*t*_ indicate if the dog ate everything from the snuffle mat and tray respectively. The average activity is an average of the daily step counts for every day that the individual participated in the study. Two dogs (Blaze and Sasha) had technical errors with their activity collars, therefore step data was not available for those participants.

Dog	Breed	Sex	Age	BCS	*IC*_*s*_	*IN*_*s*_	*E*_*s*_	*E*_*t*_	Average activity	Preference for CFL?	Willing to CFL?
Archie	Mixed Breed	M	9	3.00	0.1	0.7	Y	Y	13,388	Freeloader	Y
Barley	Mixed Breed	M	3	2.50	0.4	1	Y	Y	22,419	No preference	Y
Benji	Mixed Breed	M	1	3.00	0.1	0.2	Y	Y	23,480	Freeloader	Y
Blaze	Mixed Breed	F	4	3.60	0.1	0.9	N	Y	N/A	Freeloader	Y
Cece1	Havanese	F	6	2.60	0.2	0.5	Y	Y	18,349	Freeloader	Y
Cece2	Mixed Breed	F	2	2.50	0	0.2	N	N	29,403	Freeloader	N
Chacha	Mixed Breed	F	2	4.00	0.6	1.3	N	N	16,771	No preference	Y
Chippie	Shih Tzu	M	7	3.00	0.3	0.6	N	Y	12,066	No preference	Y
Coco	Mixed Breed	F	1	3.00	0.4	1.6	N	Y	12,582	No preference	Y
Dexter	Mixed Breed	M	7	4.00	0.6	0.63	N	N	17,608	No preference	Y
Ellie	Bulldog	F	6	3.00	0	1.8	N	Y	13,304	Freeloader	N
Frankie	Mixed Breed	M	9	2.30	0.2	0.5	Y	Y	5797	Freeloader	Y
Hurley	Soft-Coated Wheaten Terrier	M	4	3.00	0.1	1.7	N	Y	26,728	Freeloader	Y
Karma	Mixed Breed	F	4	4.00	0.8	0.9	Y	Y	11,570	Contrafreeloader	Y
Kazoo	Mixed Breed	M	3	3.00	0.5	1.3	Y	Y	30,231	No preference	Y
Lenny	Shih Tzu	F	5	3.00	0.3	0.6	N	Y	19,155	No preference	Y
Lilly	Rottweiler	F	2	3.00	0	0.3	Y	Y	30,002	Freeloader	N
Loubie	Pug	F	1	3.00	0.7	2.9	Y	Y	8217	No preference	Y
Louie	Yorkshire Terrier	M	4	1.90	0	1.7	N	N	24,771	Freeloader	N
Lucy	Mixed Breed	F	9	3.00	0.3	0.11	N	N	10,134	No preference	Y
Luna	Golden Retriever	F	8	3.50	0.1	1.4	N	Y	3211	Freeloader	Y
Mak	Labrador Retriever	M	2	3.50	0.2	0.9	N	N	17,378	Freeloader	Y
Nellie	Mixed Breed	F	2	3.60	0.2	0.9	Y	Y	13,297	Freeloader	Y
Nessie	Mixed Breed	F	2	3.00	0.6	2.6	N	N	12,350	No preference	Y
Pearl	Mixed Breed	F	6	3.00	0.3	0.7	N	N	19,455	No preference	Y
Penny	Mixed Breed	F	1	3.80	0.2	0.11	N	N	20,369	Freeloader	Y
Pogacs	Puli	F	11	3.25	0.1	1.4	N	Y	7,093	Freeloader	Y
Pookie	Jack Russell Terrier	F	6	2.00	0	0.7	N	Y	29,506	Freeloader	N
Sadie	Mixed Breed	F	1	3.00	0	0.6	Y	Y	22,220	Freeloader	N
Sasha	Mixed Breed	F	7	3.00	0.2	0.3	N	N	N/A	Freeloader	Y
Simon	Mixed Breed	M	2	3.00	0.6	1.6	Y	Y	16,796	No preference	Y
Sully	Golden Retriever	M	1	2.70	0.2	0.8	N	N	19,715	Freeloader	Y
Theo	Golden Retriever	M	2	3.00	0.2	1.2	N	N	13,018	Freeloader	Y
Toby	Mixed Breed	M	9	3.00	0.5	1.3	N	N	10,735	No preference	Y
Turner	Golden Retriever	M	4	2.50	0	0.4	Y	Y	1099	Freeloader	N
Vienna	Dachshund	F	4	3.00	0.56	1.56	N	N	18,646	No preference	Y
Wilson	Miniature Schnauzer	M	1	3.00	0	0.8	Y	Y	35,304	Freeloader	N
Zoe	Mixed Breed	F	2	3.00	0.3	0.6	N	N	12,842	No preference	Y

**Figure 2:**
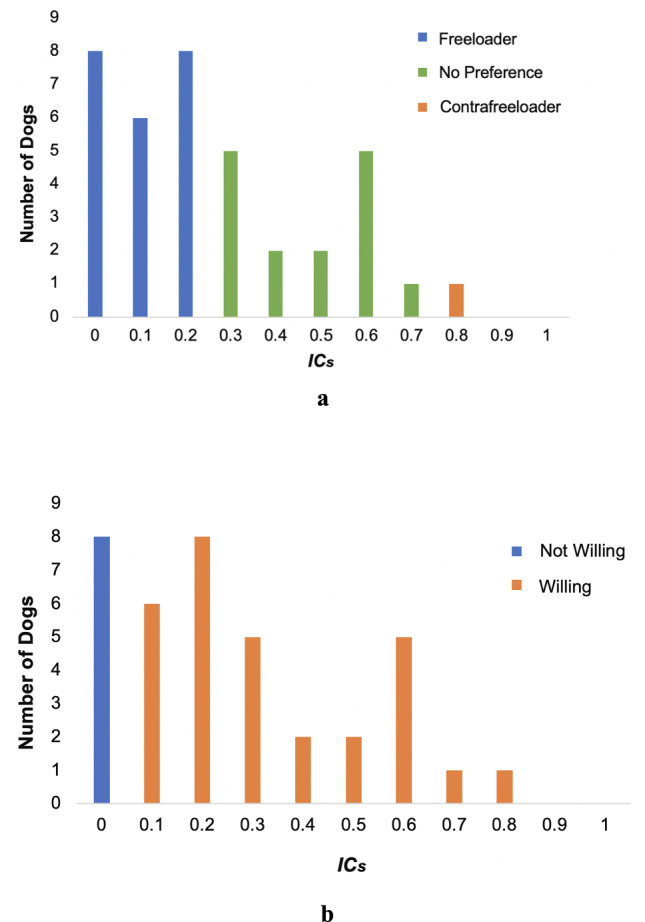
(**a**) Number of dogs with a preference to contrafreeload using *IC*_*s*_. *IC*_*s*_ scores were rounded to the nearest tenth for the purpose of this chart. (**b**) Number of dogs with a willingness to contrafreeload using *IC*_*s*_. *IC*_*s*_ scores were rounded to the nearest tenth for the purpose of this chart.

In evaluating the *IC*_*s*_ values for preference, only one of the dogs (Karma) had an *IC*_*s*_ value of 0.8, 15 dogs had *IC*_*s*_ values greater than 0.2, but less than 0.8, and 22 dogs had *IC*_*s*_ values of less than 0.2 (M = 0.262, SD = 0.228) (Fig. 2a). In evaluating the *IC*_*s*_ values for willingness, 30 of the dogs had an *IC*_*s*_ value greater than 0, and eight dogs had *IC*_*s*_ values equal to 0 (Fig. 2b). The full model for *IC*_*s*_ was not statistically significant (*F* (5,29) = 2.223, *p* = 0.079, η^2^p = 0.277) (Table 3). However, the individual term, BCS, was significant. Thus, a model reduction was conducted in which only BCS was considered as an effect. This reduced model, $IC_s \sim 1+BCS$, was statistically significant (*F* (1,36) = 7.72, *p* = 0.009, η^2^p = 0.177) (Table 4).

**Table 3:** Results of the full model with *IC*_*s*_ score as the response including the effects of sex, experience, age, activity and BCS (estimates, standard errors, confidence intervals, and test results).

Term	Estimate	SE	Lower CI	Upper CI	β	df	t	p
Intercept	0.25744	0.0454	0.1645	0.3503	0.0000	29	5.667	< 0.001
Sex	0.01040	0.0753	− 0.1436	0.1644	0.0444	29	0.138	0.891
Experience	0.05951	0.0992	− 0.1434	0.2625	0.2543	29	0.600	0.553
Age	− 0.00571	0.0152	− 0.0368	0.0254	− 0.0665	29	− 0.376	0.710
Activity	− 7.89e − 6	5.41e − 6	− 1.90e − 5	3.17e − 6	− 0.2695	29	− 1.459	0.155
BCS	0.21671	0.0850	0.0428	0.3906	0.4546	29	2.548	0.016

**Table 4:** Results of the reduced model with *IC*_*s*_ score as the response including the effect of BCS (estimates, standard errors, confidence intervals, and test results).

Term	Estimate	SE	Lower CI	Upper CI	β	df	t	p
Intercept	0.262	0.0340	0.1931	0.331	0.000	36	7.70	< 0.001
BCS	0.199	0.0716	0.0538	0.344	0.420	36	2.78	0.009

In evaluating *IN*_*s*_ values, all 38 dogs had a score greater than 0 (M = 0.982 per trial; SD = 0.643). The overall model with *IN*_*s*_ was not statistically significant (*F* (5,29) = 1.231, *p* = 0.320, η^2^p = 0.175). None of the individual terms in the model with *IN*_*s*_ had a *p* value < 0.05 (Table 5).

**Table 5:** Results of the full model with *IN*_*s*_ score as the response including the effects of sex, experience, age, activity and BCS (estimates, standard errors, confidence intervals, and test results).

Term	Estimate	SE	Lower CI	Upper CI	β	df	t	p
Model	0.87159	0.1361	0.593	1.1500	0.0000	29	6.4026	< 0.001
Sex	− 0.01710	0.2256	− 0.479	0.4444	− 0.02605	29	− 0.0758	0.940
Experience	0.43909	0.2974	− 0.169	1.0473	0.66876	29	1.4766	0.151
Age	− 0.06821	0.0456	− 0.161	0.0250	− 0.28277	29	− 1.4962	0.145
Activity	− 3.30e − 5	1.62e − 5	− 6.62e − 5	1.26e − 7	− 0.40204	29	− 2.0375	0.051
BCS	− 0.00729	0.2548	− 0.528	0.5139	− 0.00545	29	− 0.0286	0.977

## Discussion

The present study evaluated if dogs demonstrate a preference and/or a willingness to work for food, also known as contrafreeloading. To our knowledge, this study is the first to evaluate contrafreeloading behavior in dogs. Overall, the results indicate that pet dogs show a *willingness* but not a *preference* to contrafreeload, to work for food when freely available food is present. While the majority of dogs were *willing* to contrafreeload, only one dog demonstrated a *preference* to contrafreeload, and most would in fact be considered freeloaders.

These results align with the findings from Delgado et al.^10^ and other previously discussed literature that animals generally demonstrate a *willingness* to contrafreeload but a *preference* to do so can be impacted by many factors. For example, research suggests that contrafreeloading levels are lower when information about a food source can be visually assessed^20^. Since the food on the tray was visually available to the dogs, they likely had no biological need to find food elsewhere in the snuffle mat. Hunger may also have been a factor; dogs in this study were not fed ad libitum, and many individuals ate all the food that was offered in both feeders during every feeding trial. The weak contrafreeloading behavior observed in both this study and in cats^10^ supports the information primacy model for contrafreeloading^7^. Companion animals likely have very little need to prioritize exploration and foraging, as they are typically given resources by the owners and live in stable environments, and therefore would not be required to look for food sources in a changing environment. Future research should assess if other canids, such as gray wolves (*Canis lupus*) and dingoes (*Canis lupus dingo*) demonstrate contrafreeloading behavior due to sharing a common ancestor with the domestic dog. Alternatively, do free-ranging domestic dogs contrafreeload, and if not, does the domestic dog’s relationship with humans influence their willingness or preference to contrafreeload?

The second research question posed in this study was to evaluate the impact of physical fitness on a dog’s willingness to contrafreeload. BCS did have a significant effect on *IC*_*s*_. Dogs with higher BCS scores (owner-reported) demonstrated a greater willingness to contrafreeload. This result is consistent with the literature that suggested contrafreeloading behavior is said to increase when animals are not as hungry^13^. Since dogs with greater BCS scores might not be as hungry, it makes sense that these dogs would display more contrafreeloading behavior. On the other hand, previous studies have found a correlation between exploration and contrafreeloading behavior^20^. However, the present study did not find an effect of activity on contrafreeloading behavior. Future studies could consider utilizing an alternative measure of exploration. Since most of our sample lived in New York City, it is plausible that dog activity is more greatly influenced by exercise opportunities offered by the owner than individual dog differences in desired amounts of exploration or activity.

The third and final research question posed in this study was to determine if a dog’s contrafreeloading behavior was associated with prior experience with food enrichment devices. Although there is evidence that prior experience with an earned reinforcer affects contrafreeloading behavior^43^, prior enrichment experience (owner-reported) did not have a significant effect on *IC*_*s*_ and *IN*_*s*_ values. Within pet dog populations, future research should aim to determine if the limited contrafreeloading behavior observed in this study could be related to the effort needed (i.e., are dogs more willing to work for easier or more difficult puzzles). It would be worthwhile to see if dogs contrafreeload more when presented with an alternative style of food puzzle (such as a standard slow feeder or Nina Ottosson style puzzles) as opposed to a snuffle mat.

This study was not without its limitations. Inclusion criteria required dogs who primarily ate a dry food based diet. This was due to the likelihood of wet food sticking to the fabric of the snuffle mat. Thus, dogs eating certain diets may not have been represented in the present investigation. Another limitation is the lack of standardization across feeding times, both within and between participants. To be less disruptive to the dog’s normal routine, we did not standardize the times the dog was fed and how much additional food the dogs got throughout the day. Therefore, a wide range of hunger levels may have been represented in this study. Additionally, due to the nature of community science, additional errors are to be expected. For example, since BCS was owner reported, it is possible that owners would underestimate or overestimate their dog’s body condition. It is possible that there could be some variability in our results due to environments not being equivalent for all dogs. Finally, another limitation is that only certain breeds, and many mixed breeds were included in the sample studied. Given the diversity in domestic dog breeds, subsequent studies should evaluate the impact of breed differences and breed types on contrafreeloading behavior, as prior research has shown that some breeds are genetically more prone to over-eating and obesity^58^. Thus, the results are unlikely to be broadly generalizable to all dogs and particular attention to individuals should be considered if these results are to be applied to other contexts.

In conclusion, contrafreeloading represents an interesting behavior to study as it opposes a basic tenet of animal learning and evolutionary theory. Research shows that many domesticated animals do have a preference for this behavior, however, the present study suggests dogs may not have a *preference* to contrafreeload, but might be *willing*. These findings have implications for enrichment practices. However, it should be noted that the results of the study do not suggest that snuffle mats are not enriching for dogs. What the results do suggest is that when faced with the choice between freely available food from a tray and effort-required food from a snuffle mat, dogs generally prefer to approach the freely available food first but are sometimes willing to approach the earned food feeder first. Just because a dog might not be more likely to choose freely available food over earned food, it does not negate the value of environmental enrichment for dog welfare. Although our results do not show a preference for contrafreeloading, it is possible that some individual dogs do prefer to contrafreeload. Therefore, it is important that owners understand their own dog’s preferences for enrichment to best support their welfare.

### Supplementary Information

Supplementary Information 1.

Supplementary Information 2.

## Data Availability

All data generated or analyzed in this study are included in this article (and its Supplementary Information files).
