# Supplementary material for: Domestic pet dogs (Canis lupus familiaris) do not show a preference to contrafreeload, but are willing
Source: Sci Rep. 2024 Jan 15;14:1314. doi: 10.1038/s41598-024-51663-x (PMC10789754; doi:10.1038/s41598-024-51663-x)
Supplement: Supplementary file 1 — Supplementary Information 1. [file 41598_2024_51663_MOESM1_ESM.pdf]

Supplementary Material S1

Thank you for your interest in participating in the Hunter College Thinking Dog Center's community science project "Eating Styles"! To explore scientific questions about which eating styles dogs prefer, we need your help! In summary, this study asks you to offer your dog two feeding styles, 10 times, over the span of 5-10 days. Participation can be incorporated into your average day with your pet.

If you are interested in participating in this study with your dog, please take a moment to fill out this questionnaire. The questionnaire takes about 15 minutes, and it's important that you fill it out as completely as possible so that we can get to know your dog. This will allow us to determine if this study is suitable for them to participate in. If you would like to register multiple pets, please fill out the questionnaire for each dog independently.

If your dog is selected to participate in this study, you will be asked to join us at the Thinking Dog Center or Hunter College to pick up all necessary equipment. Our research team will provide you with regular guidance throughout your dog's participation in this study. The study concludes once you have 1) completed this questionnaire and 2) been selected to participate and 3) uploaded 10 feeding videos of your dog. Once completed, your dog will be awarded a participation certificate for their time, a Fi Collar activity tracker, and entered to win a free year subscription to the Fi - GPS Dog Tracker app.

Any personal information (name and e-mail address) about the owner provided as a part of this questionnaire will be disclosed to our research team for the sole purpose of contacting you throughout the project, or for following up with raffle information. Any questions about your pet will be used to determine suitability in the study as well as for research purposes. Please keep in mind that you can withdraw your pet and your contact information from our studies at any time and that the submission of this form does not obligate you to participate in any way nor does it guarantee your dog's placement in this study.

By agreeing to this statement, I understand that I have volunteered myself and my dog to be involved in research at the Thinking Dog Center. By participating in the project, Eating Styles, I acknowledge and consent to Fi Smart Dog Collar sharing information pertaining to my dog's activity with the researchers at the Thinking Dog Center. Information shared with the researchers will not include location data, but will include information such as the number of steps, activity summaries and sleeping patterns during the duration of the study. Email addresses will be kept and stored by Fi Smart Dog Collar. Upon your dog's completion of the project, no additional information will be shared with researchers at the Thinking Dog Center.

This study has been approved by the CUNY Hunter College Institutional Animal Care and Use Committee (SEB-FreeloadingDogs 2/25).

Have a question? E-mail us at tdceatingstyles@gmail.com

- ◯ Yes, I would like to sign-up my dog to participate in the study Eating Styles.

About Your Dog

Your (owner) name:*

Your email address:*

Your phone/mobile number:*

Your preferred method of contact:*

- ◯ Email
- ◯ Phone

Your zip code (US postal code):*

What is your dog's name?*

What is your dog's approximate date of birth (Month/Year)? If you do not know, please use your best estimate.*

	Month	Year (XXXX)
Please select:		

What is your dog's sex?*

- Male
- Female

Is your dog a purebred or mixed breed?*

- Purebred
- Mixed breed

What breed is your dog?*

What breed(s)? If you're not sure, please give your best guess.*

Is you dog desexed (e.g. spayed or neutered)?*

- Yes
- No

Does food motivate your dog?*

- Yes
- No

Do toys motivate your dog?*

- Yes
- No

Does your dog have any food allergies or dietary restrictions?*

- Yes
- No

Provide optional information about your dog's food allergies and dietary restrictions:*

Does your dog have a history of fear, anxiety, or aggression (e.g. biting, lunging, fear of men)?*

- Yes
- No

Provide optional information about your dog s history of fear, anxiety, and/or aggression:*

Has your dog ever injured (e.g., bitten) a person?*

- Yes
- No
- Not sure

Provide optional information about the resulting injury:*

Are you interested in getting involved in future studies at the Thinking Dog Center?*

- Yes, I am interested in bringing my dog to participate at the center on West 36th Street between Broadway and 7th Avenue.
- Yes, I am interested in participating in online or remote studies with my dog.
- Yes, I am interested in bringing my dog to participate at the center on West 36th Street AND participating in online or remote studies with my dog.
- No, I am only interested in having my dog participate in this study.

We love sharing pictures of our participants on social media! If your dog has an Instagram, let us know their handle below so that we can tag them in photos!

Default Question Block

Do you have a camera-enabled smartphone?*

- Yes
- No

Is your dog afraid of new objects?*

- Yes
- No
- Other (please explain):

To the best of your knowledge, does your pet have any visual disabilities (e.g., blindness or visual impairment)?*

- Yes (please explain):
- No

Does your dog live in a multi-pet household?*

- Yes
- No

Within the next six weeks, will your dog live in a multi-pet household?*

- Yes
- No

Do your pets eat meals separately (e.g., in separate rooms, separate kennels)?*

- Yes
- No

What best describes your pet's feeding setup?*

- ◯ The pet is fed (e.g., placing a bowl) by me or another owner at most meal times
- ◯ Food is left out for the pet to consume at their leisure
- ◯ The pet is fed by an automatic feeder
- ◯ A combination of these (feel free to detail) ______
- ◯ Other (please explain): ______

On average, how often is your dog fed daily? Please only report main feedings (Do not include treats given throughout the day).*

[dropdown]

What consistency of food does your pet mostly eat during their meals? Select all that apply.*

- ☐ Dry food (anything without moisture, e.g., kibble, dehydrated)
- ☐ Wet food (anything with moisture, e.g., canned, raw)
- ☐ Other (please explain): ______

How much food, in cups, does your dog eat per meal?*

______

What is your dog's weight in pounds (lb)?*

______

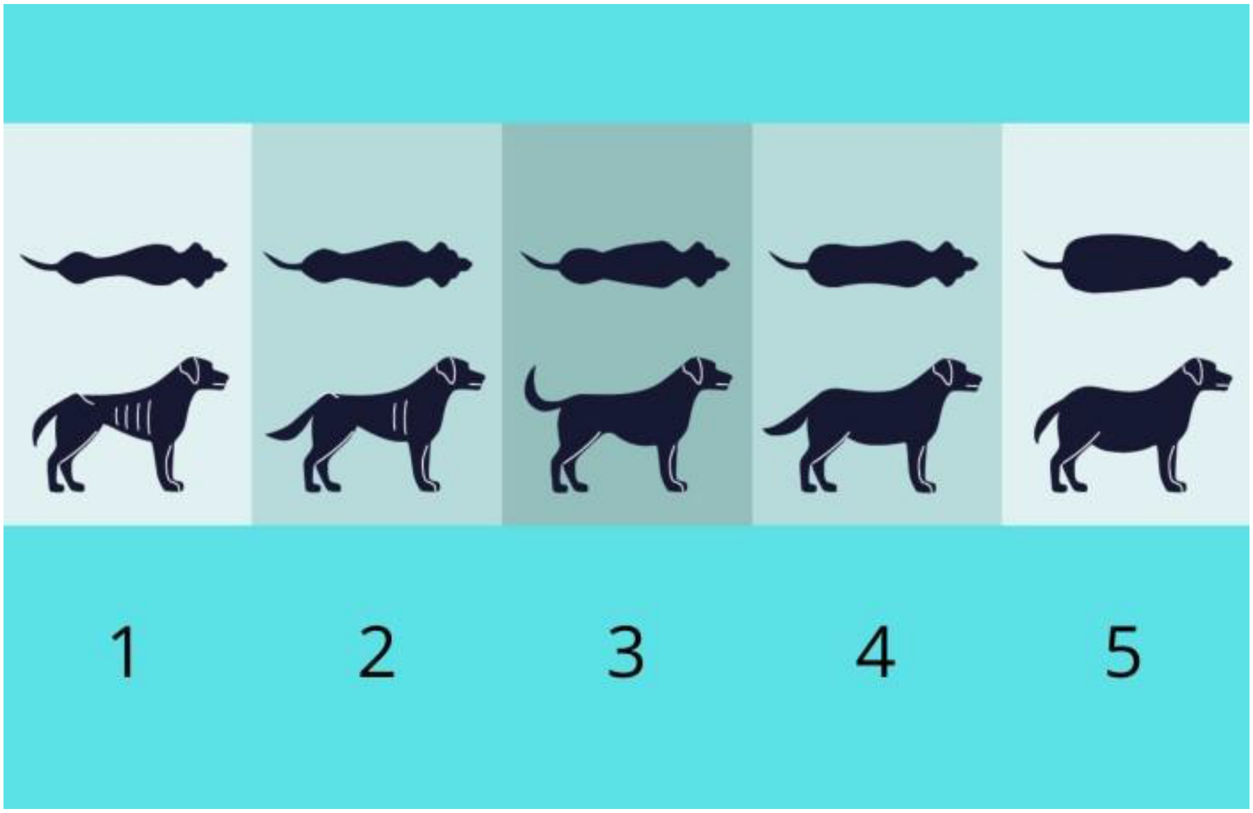

Using the above chart, what is your dog's body condition score?*

	1	2	3	4	5
Body Condition Score					

Has your dog had any previous experience with a food enrichment toy (e.g. Nina Ottosson puzzle, slow feeder, wobble feeder, snuffle mat, licking mat)?*

○ Yes

○ No

○ Unsure

What types of food enrichment toys does your dog have previous experience with? Please select all that apply.*

☐ Slow feeder

☐ Wobble feeder

☐ Snuffle mat

☐ Nina Ottosson-like puzzle

☐ Licking mat

☐ Kong

☐ Treat dispensing ball

☐ Other (please explain): ______

How often does your dog experience each type of enrichment toy?*

	Every day	Every 2-3 days	Every week	Every 2-3 weeks	Every month	Every 2-3 months	Less than every 2-3 months
» Slow feeder	○	○	○	○	○	○	○
» Wobble feeder	○	○	○	○	○	○	○
» Snuffle mat	○	○	○	○	○	○	○
» Nina Ottosson-like puzzle	○	○	○	○	○	○	○
» Licking mat	○	○	○	○	○	○	○
» Kong	○	○	○	○	○	○	○
	○	○	○	○	○	○	○
______	○	○	○	○	○	○	○

» Treat dispensing ball
» Other (please explain):

Does your dog wear a Fi Smart Dog Collar?*

◯ Yes

◯ No

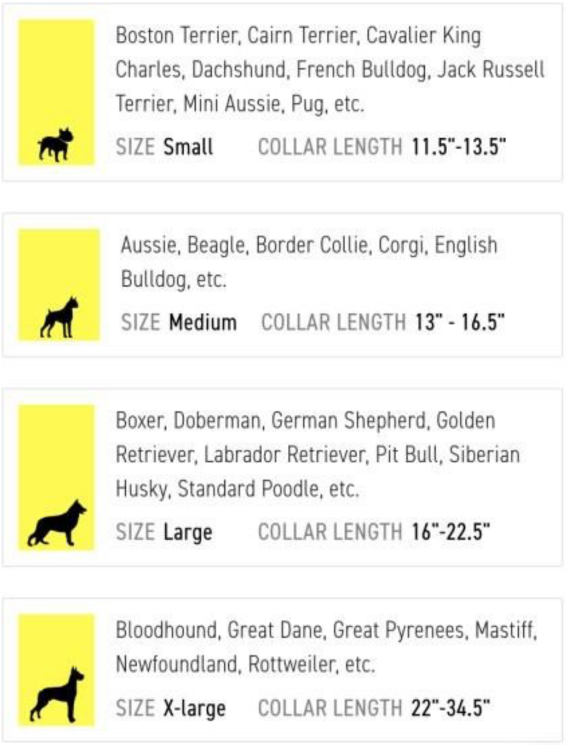

What size Fi Collar would best fit your dog?*

- ◯ Small
- ◯ Medium
- ◯ Large
- ◯ X-large

Please rank your collar color preference, with 1 being your first choice and 4 being your last choice.* Note: We will try our best to give you your first choice color, however this is not guaranteed.

- Yellow ______
- Gray ______
- Blue ______
- Pink Ombre ______

I, the owner (as identified below), certify and consent to the following:*

- ☐ I am 18 years of age or older.
- ☐ I am voluntarily participating in the present study with my dog, and I agree that research data provided by me or with my permission during the project may be included in a thesis, presented at conferences and published on the condition that neither my name nor any other identifying information is used.
- ☐ I understand that even though I have volunteered myself and my dog to be involved in this project, I can withdraw from the study at any time, and can withdraw my data, for any reason, up to four weeks following the completion of our participation in the research. Further, in withdrawing from the study, I can request that no information from my involvement be used.

Supplementary Material S2

Thank you for volunteering your dog to participate in this study! Here is your guide to completing this community science study. Please read the following instructions carefully. **If you have any questions, do not hesitate to reach out to Liza and our Team at tdceatingstyles@gmail.com.**

If you have not already done so, join our study's Facebook group (Thinking Dog Center Eating Styles Participant Group) and introduce yourself and your dog! This group is an open and private space to ask questions about the study and share photos!

Here's what is included in your kit:

**Item Name**	**Item Picture**
**The Tray**	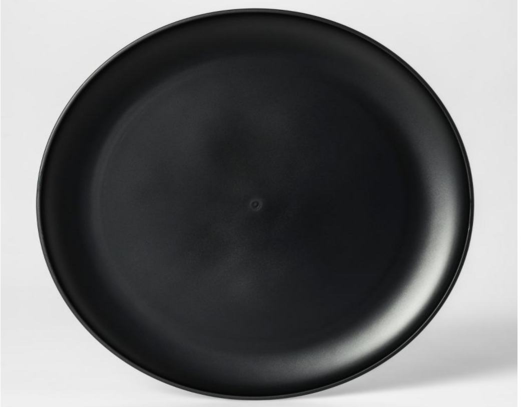
**The Snuffle Mat**	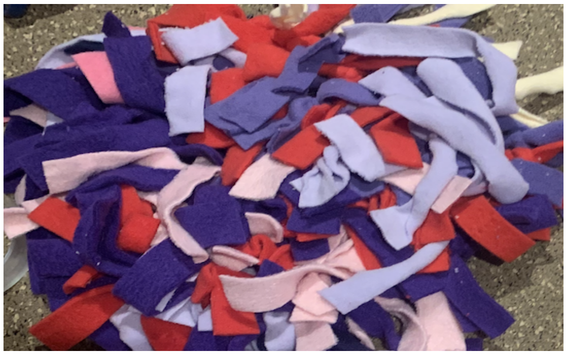
**A Scale (set to grams!)**	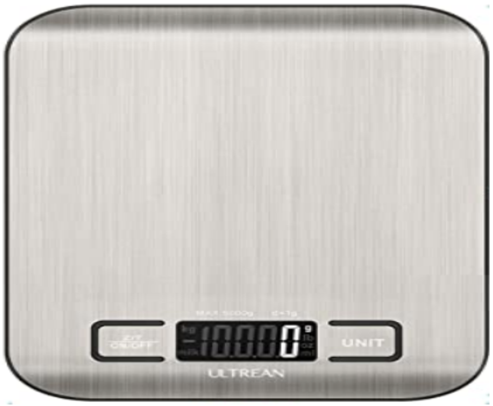

**Fi Activity Tracker Collar**	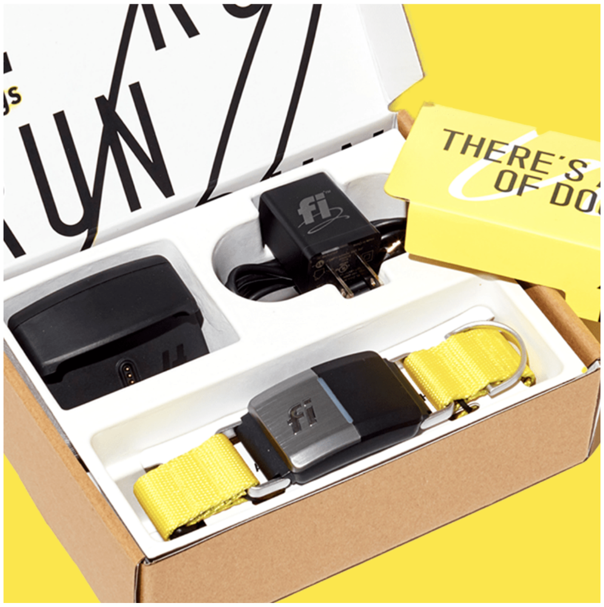
**Stickers**	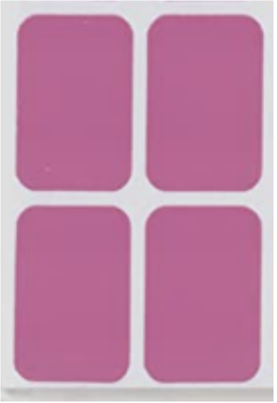

Here's what else you will need:

1. **Your dog!**
2. **Your dog's dry kibble/food (at each feeding this should constitute their full meal)**
3. **A camera-enabled smartphone**
4. **A measuring tape or smartphone measure application**
5. **Measuring utensils**
6. **A cup/bowl**

Setting Up Your Fi Collar Activity Tracker Collar:

As a part of the study, your dog will wear a Fi Activity collar. Detailed set-up instructions can be found with your device, however, additional instructions can be found here.

- Set-up your Base and charge your collar! Most collars do not come pre-charged. Please prepare to set-up your collar at least 6 hours before you plan to begin the study at home.

- Fit the collar to your dog’s neck.
- Download the Fi Application.
- For the duration of the study, please do not purchase the GPS subscription. Once your dog has completed the study and you have received the okay from the Eating Styles Team, you are welcome to purchase the GPS subscription if you’d like!
- **IMPORTANT: YOUR DOG SHOULD WEAR THE COLLAR 24/7 FOR THE DURATION OF THE STUDY.** However, if you do not feel comfortable leaving the collar on when your dog is unattended, feel free to take it off for those periods of time.

Creating Your Feeding Set-Up:

Before getting started with the feedings, find a spot on the floor that would be a good space for the feedings. Ensure that the chosen spot has enough space for your dog to eat comfortably, as well as enough space to put the camera 4 feet in front of the feeding set up.

- Once you find the perfect spot, measure two feet, preferably about four feet from a wall. Put one sticker at the 0” mark, and another at 24” mark. These stickers will be where you place the feeders.

- Measure 4 feet from the middle of the previously placed two stickers (the feeding setup). Mark this spot with another sticker. This spot is where your phone will be set up while filming.

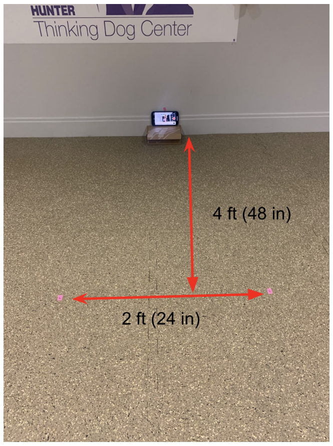

- Measure 6 feet from in between the feeder stickers, in the opposite direction from the phone spot. This will be your dog’s starting position. Mark this spot with a sticker.

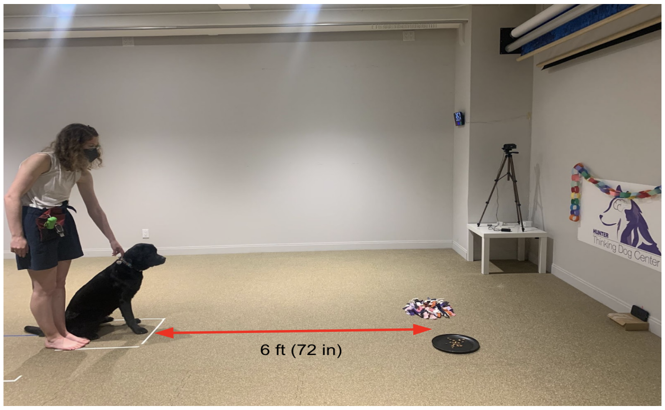

- You now have your feeding set-up prepped!

Starting the Experiment:

Once you have set up the feeding space and collar, here is how to get started with the experiment!

1. Gather your dog's food!

2. Measure out the predetermined amounts of the dog's food for each feeder (e.g., the Tray and the Snuffle Mat). See below for where the amount of food will be found on the data sheet.

Feeding Name/#	Feeder Placement (dog's perspective)	Date/Time	Tray	Snuffle
**Sample**	**Tray: Right**<br>**Snuffle: Left**	**7/14/22, 1:30pm**	**1 cup**<br>weight before<br>__________g<br>weight after<br>__________g	**2 cups**<br>weight before<br>__________g<br>weight after<br>__________g

3. Place each portion of food into separate cups/bowls and weigh the portions of the food separately on the scale in grams (double check your scale as it has multiple modes!). Record these values in the given table in the space indicated below.

Feeding Name/#	Feeder Placement (dog's perspective)	Date/Time	Tray	Snuffle
**Sample**	**Tray: Right**<br>**Snuffle: Left**	**7/14/22, 1:30pm**	**1 cup**<br>weight before<br>__________g<br>weight after<br>__________g	**2 cups**<br>weight before<br>__________g<br>weight after<br>__________g

Pro-Tip: You can place the cup/bowl on the scale and reset the scale to zero. Then when you add your dry food, you do not need to subtract the weight of the bowl!

4. Add the portioned out amounts onto the tray and snuffle mat as determined above in Step 3.

   How to fill the snuffle mat: Place the portioned amount into the snuffle mat by separating the pieces of fabric and dropping the pieces of food between them, ensuring the food is hidden.

5. Record the date and time the feeding starts in the table.

6. Set your camera up landscape (horizontally) at the camera sticker marker and begin recording the video with the camera directed at your dog.

   Pro-Tip: You can use the Fi Collar box to prop up your phone (see photo).

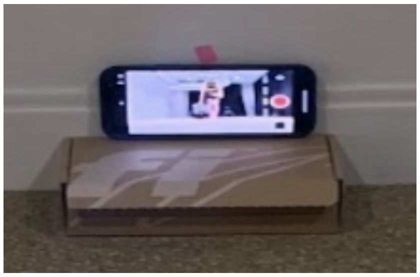

7. Be sure to use the data sheet to check which feeder needs to be on the Left or Right (based on the dog's perspective).

8. Here comes the important human part!
   a. Place the feeders on the floor, making sure the feeders are centered on the sticker marks placed on the floor for the feeding setup.
   b. Your dog cannot be loose while you are putting the trays down. Your dog must either be in another room or held back by another person at the starting position.
   c. If you are placing down the trays in front of your dog, you must place them on their predetermined spots at the same time.

.

REMEMBER: Your dog cannot be loose while you are putting the trays down. Your dog must either be in another room or held back by another person.

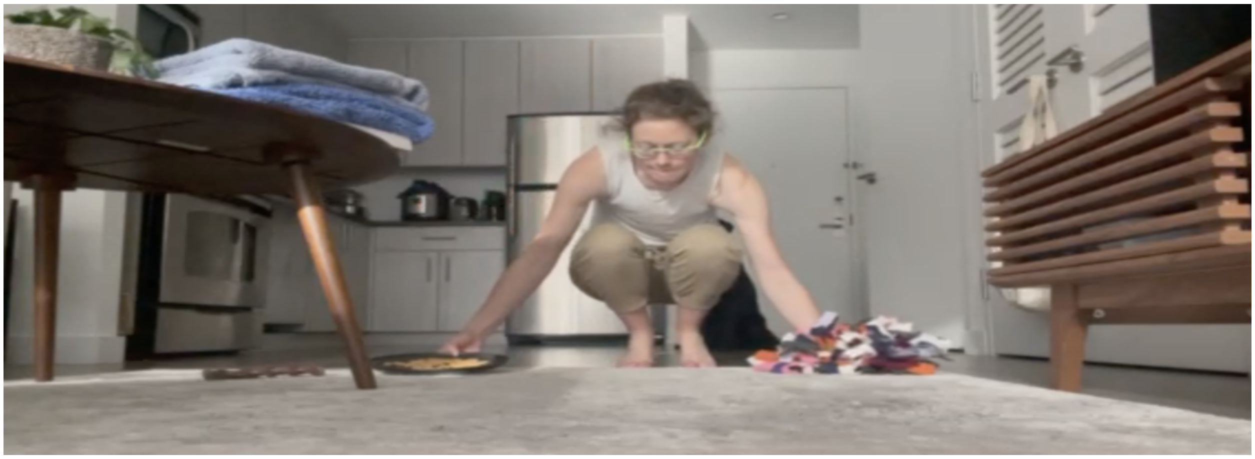

9. When placing the feeders on the floor, press the tray and snuffle mat down so that they are stuck to your floor. This helps to minimize your pup from moving them around!

10. Bring your dog to the previously marked starting position, centered between the two feeding trays.

11. Release your dog from the starting position, allowing them to approach the feeders. See video for details: https://drive.google.com/drive/folders/1NF9ypItDEB0-mLc6rgIrDsBbeLdj6Rp5?usp=sharing.

12. Now let your dog eat uninterrupted! Please stand back and try not to distract or interact with your dog until they have finished eating. Use your best judgment, if you think your dog is behaving inappropriately with one of the feeders, or another item in your home, please interrupt them. We do not want your dog to get into anything they shouldn't!

13. Stand still and continue recording until your dog walks away from both feeders for a minimum of 10 seconds or after 15 minutes have elapsed (whichever happens first).

14. Stop recording and pick up both feeders from off the floor.

15. If there is food left in the feeder(s), weigh the food left in each feeder separately (in the same manner as above), and record the weight of the food in the corresponding column on your data sheet, as shown below. If your dog has eaten everything, please write "N/A".

**Feeding Name/#**	**Feeder Placement (dog's perspective)**	**Date/Time**	**Tray**	**Snuffle**
**Sample**	**Tray: Right**<br>**Snuffle: Left**	**7/14/22, 1:30pm**	**1 cup**<br>weight before<br>__________g<br>weight after<br>__________g	**2 cups**<br>weight before<br>__________g<br>weight after<br>__________g

16. You may offer any remaining food from the feeders to your dog in their regular feeding bowl/device.

17. Now that your dog has finished their feeding trial, please email the video to Liza (tdceatingstyles@gmail.com) with the subject line [Dog's Name_Owner Last Name] [Session #/Feeding #] (e.g., Sadie_Vorva Acclim 1). We recommend using mail drop. Because our Team needs to follow along with your dog's feedings in real-time, please be sure to upload your dog's feeding right after they've finished. See below for an example email.

    tdceatingstyles@gmail.com

    Ellie_Rothkoff Acclim 4

    Hello! I have attached Ellie's 4th acclimation session. Please see below for the attached video!

    Best,
    Liza

    NOTE: If you would prefer another method of submitting your videos, you can also upload your videos through Dropbox (using this link: https://www.dropbox.com/request/mkoTk5mFAi3ogw8spoVp) or through Google Drive. Please be sure to specify which Session #/Feeding # you are submitting.

18. Repeat the above steps for every feeding session.

19. After your first feeding session (Acclim 1), be sure to send our team a photo of your data sheet so that we can ensure that the first entry is filled out correctly.

20. At the end of the study, send a copy of your filled out data sheet to tdceatingstyles@gmail.com.

Supplementary Material S3

Dog Name				
Food (cups) at each meal	**100%**			
**Feeding Name/#**	**Feeder Placement (dog's perspective)**	**Date/Time**	**Tray**	**Snuffle**
**Sample**	**Tray: Right**<br>**Snuffle: Left**	**7/14/22, 1:30pm**	**1 cup**<br>weight before 235 g<br>weight after N/A g	**2 cups** weight before 470 g<br>weight after 200 g
**Acclim 1**	**Tray: Right**<br>**Snuffle: Left**		**75%**<br>weight before ___________g<br>weight after ___________g	**25%**<br>weight before ___________g<br>weight after ___________g
**Acclim 2**	**Tray: Left**<br>**Snuffle: Right**		**50%**<br>weight before ___________g<br>weight after ___________g	**50%**<br>weight before ___________g<br>weight after ___________g
**Acclim 3**	**Tray: Left**<br>**Snuffle: Right**		**50%**<br>weight before ___________g<br>weight after ___________g	**50%**<br>weight before ___________g<br>weight after ___________g
**Acclim 4**	**Tray: Right**<br>**Snuffle: Left**		**25%**<br>weight before ___________g<br>weight after ___________g	**75%**<br>weight before ___________g<br>weight after ___________g

STOP!

Before moving on to the next phase of the study, you need to email our Team your Acclim 4 video. Once you have obtained approval to move on (within 24 hours) you can continue!

Dog Name				
Food (cups) at each meal	**100%**			
**Feeding Name/#**	**Feeder Placement (dog's perspective)**	**Date/Time**	**Tray**	**Snuffle**
**Test 1**	Tray L<br>Snuffle R		**50%**<br>weight before<br>___________g<br>weight after<br>___________g	**50%**<br>weight before<br>___________g<br>weight after<br>___________g
**Test 2**	Tray R<br>Snuffle L		**50%**<br>weight before<br>___________g<br>weight after<br>___________g	**50%**<br>weight before<br>___________g<br>weight after<br>___________g
**Test 3**	Tray L<br>Snuffle R		**50%**<br>weight before<br>___________g<br>weight after<br>___________g	**50%**<br>weight before<br>___________g<br>weight after<br>___________g
**Test 4**	Tray L<br>Snuffle R		**50%**<br>weight before<br>___________g<br>weight after<br>___________g	**50%**<br>weight before<br>___________g<br>weight after<br>___________g
**Test 5**	Tray R<br>Snuffle L		**50%**<br>weight before<br>___________g<br>weight after<br>___________g	**50%**<br>weight before<br>___________g<br>weight after<br>___________g
**Test 6**	Tray R<br>Snuffle L		**50%**<br>weight before<br>___________g<br>weight after<br>___________g	**50%**<br>weight before<br>___________g<br>weight after<br>___________g

**Test 7**	Tray L<br>Snuffle R		**50%**<br>weight before<br>___________g<br>weight after<br>___________g	**50%**<br>weight before<br>___________g<br>weight after<br>___________g
**Test 8**	Tray R<br>Snuffle L		**50%**<br>weight before<br>___________g<br>weight after<br>___________g	**50%**<br>weight before<br>___________g<br>weight after<br>___________g
**Test 9**	Tray L<br>Snuffle R		**50%**<br>weight before<br>___________g<br>weight after<br>___________g	**50%**<br>weight before<br>___________g<br>weight after<br>___________g
**Test 10**	Tray R<br>Snuffle L		**50%**<br>weight before<br>___________g<br>weight after<br>___________g	**50%**<br>weight before<br>___________g<br>weight after<br>___________g
